# Bacteriologic and Genomic Investigation of *Bacillus anthracis* Isolated from World War II Site, China

**DOI:** 10.3201/eid3012.231520

**Published:** 2024-12

**Authors:** Yarong Wu, Yuan Yuan, Bing Yuan, Jiaxin Li, Jinglin Wang, Yujun Cui

**Affiliations:** State Key Laboratory of Pathogen and Biosecurity, Academy of Military Medical Sciences, Beijing, China

**Keywords:** *Bacillus anthracis*, bacteria, bioterrorism and preparedness, World War II, Harbin, biological warfare, genome sequencing, microbial forensics, China

## Abstract

Records suggest *Bacillus anthracis* was used in biowarfare during World War II, but evidence remains limited. We isolated *B. anthracis* from soil at the remains of a World War II–era laboratory in China. Phenotypic and genomic analyses confirmed the finding, highlighting the value of microbial forensics in biothreat investigation.

*Bacillus anthracis*, the etiologic agent of anthrax, is a gram-positive bacterium that can cause life-threatening disease among wild and domestic mammals, including humans (*1*). *B. anthracis* can form spores, enabling long-term survival under adverse conditions. Isolation of *B. anthracis* from soil stored up to 60 years has been reported previously (*2*). Because of its pathogenic features, *B. anthracis* is considered one of the most serious and threatening agents for conducting biowarfare or bioterrorism (*3*,*4*). 

In our previous study, 3 of 24 soil samples collected from a World War II (WWII) site in northeastern China (Appendix Figure 1) tested positive for *B. anthracis* using RPA/CRISPR-Cas12a, real time PCR, and metagenomic analysis (*5*). Of note, those positive samples were obtained from the site of Unit 731 (45°36′55.940″N, 126°38′33.738″E), a former bacteria laboratory run by the army of Japan (*5*). We collected an additional 24 samples from 12 collection sites located within radii of 0.5 km, 3 km, and 5 km from the remains of the WWII laboratory (Appendix Figure 2). However, we detected no trace of *B. anthracis* in the newly collected samples, implying that the positive samples we previously found likely did not originate from a local natural source. 

Using polymyxin B-lysozyme-EDTA-thallous acetate agar and API 50CHB-API 50CH biochemical reagents (BioMérieux, https://www.biomerieux.com), we successfully isolated and identified a *B. anthracis* strain (named BA20200413YY) from one of the soil samples. Morphologic, hemolytic, and biochemical analyses revealed classic *B. anthracis* phenotypes: gray, opaque, medium-sized, irregular-shaped colonies with a ground glass surface and no surrounding hemolytic rings (Figure, panel A). In addition, Gram staining revealed a bamboo-like arrangement of bacilli (Figure, panel B). We sequenced the whole genome of strain BA20200413YY using MiSeq (Illumina, https://www.illumina.com) and Sequel I (PacBio, https://www.pacb.com) platforms. We assembled reads into a complete genome totaling 5.5 Mbp, including the chromosome (5,228,177 bp) and 2 plasmids, pXO1 (181,765 bp) and pXO2 (94,821 bp). Functional genetic analysis revealed that BA20200413YY carries the 5 natural resistance genes of *B. anthracis*, which confer resistance to fosfomycin, β-lactamase, streptothricin, and macrolide, as well as 33 virulence genes associated with anthrax toxin and other exotoxins, exoenzymes, capsular synthesis, type VII secretion systems, and adherence (Appendix Table 1). 

**Figure:**
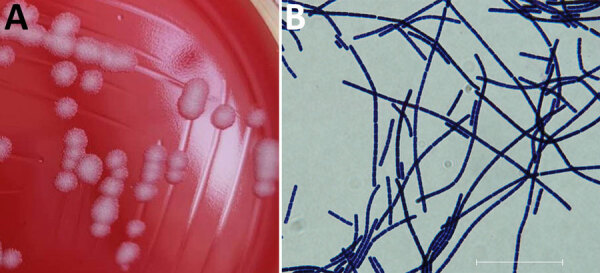
Preserved isolate from a bacteriologic and genomic investigation of *Bacillus anthracis* from World War II site, China. A) Morphological analysis of *B. anthracis* isolated on Columbia blood agar plate showing classic *B. anthracis* features: gray, opaque, medium sized, irregular-shaped colonies with a ground glass surface and no surrounding hemolytic rings. B) Gram staining showing *B. anthracis* bamboo-like arrangement. Scale bar represents 30 μm. Isolate data are available in GenBank (accession no. CPI3355587–89).

To infer the evolutionary association between BA20200413YY and other known *B. anthracis* strains, we rebuilt the phylogeny between BA20200413YY and 1,552 publicly available *B. anthracis* genomes from GenBank (https://www.ncbi.nlm.nih.gov/genbank) and Sequence Reads Archive (https://www.ncbi.nlm.nih.gov/sra) databases, based on 11,967 core genome single nucleotide polymorphisms (SNPs). Our results revealed that BA20200413YY belongs to subcluster 5.2 described elsewhere (*6*), corresponding to the classic categorization of subbranch A.Br.081 of the A.Br.002 lineage (*7*) (Appendix Figure 3, panel A). Further analysis of subcluster 5.2 strains revealed a close clustering of BA20200413YY with 9 other strains, forming a sublineage characterized by 5 lineage-specific SNPs (Appendix Table 2; Appendix Figure 3, panel B). Given the large observed genetic difference (≈35–78 SNPs) between BA20200413YY and the limited number of its close relatives (Appendix Figure 3, panel B), precisely tracing its origin was challenging. We identified 20 unique SNPs and 6 unique indels in the chromosome of BA20200413YY (Appendix Table 2). We confirmed those identifications by metagenomic sequencing of DNA extracted from the anthrax-positive soil samples from which we also isolated strain BA20200413YY. We observed no notable genomic gains or losses in either the chromosome or 2 plasmids of BA20200413YY when compared with the 9 closely related strains. 

The Fell report (*8*) described human experiments conducted at Unit 731 involving anthrax, plague, typhoid, paratyphoid A and B, shigellosis, cholera, and melioidosis using direct oral infection, infection by injection, or exposure to environmental pathogens. Moreover, the human experimental anatomy reports of anthrax (*9*) and glanders (*10*), decoded by the United States, contain information about the *B. anthracis* and *B. mallei* experiments completed at Unit 731. In this study, we isolated a strain of *B. anthracis* from soil samples collected at the former site of the bacteria laboratory of Unit 731 in Heilongjiang Province, China. Of note, all other samples collected from surrounding sites in the same region tested negative for *B. anthracis*. By analyzing the distribution of the positive samples, qualities of the isolated strain, and historical documents, we established a chain of evidence supporting the hypothesis that *B. anthracis* was misused in inhumane medical experiments and likely for developing biologic weapons during WWII. 

In conclusion, our study offers a model approach for investigating sites of historical biologic agent research related to biowarfare activities during WWII. Our findings highlight the role of microbial forensics in tracing biologic warfare and providing insights into biothreats. In addition, our results indicate that the environmental remains of hardy biologic agents pose a long-term biosecurity risk at similar WWII sites potentially contaminated with highly pathogenic biothreat agents, posing potential threats to the surrounding natural environment, and nearby humans and animals if the site is not well protected. 

Appendix Additional information from a study of bacteriologic and genomic investigation of Bacillus anthracis isolated from a World War II sit in China.
