# Supplementary material for: Bacteriologic and Genomic Investigation of Bacillus anthracis Isolated from World War II Site, China
Source: Emerg Infect Dis. 2024 Dec;30(12):2687–9. doi: 10.3201/eid3012.231520 (PMC11616637; doi:10.3201/eid3012.231520)
Supplement: Appendix — Additional information from a study of bacteriologic and genomic investigation of Bacillus anthracis isolated from a World War II sit in China. [file 23-1520-Techapp-s1.pdf]

Article DOI: https://doi.org/10.3201/eid3012.231520

EID cannot ensure accessibility for supplementary materials supplied by authors. Readers who have difficulty accessing supplementary content should contact the authors for assistance.

Bacteriologic and Genomic Investigation of *Bacillus anthracis* Isolated from World War II Site, China

Appendix

Supplementary Methods

Soil Sample collection, DNA Extraction and Detection of *B. anthracis* Genomic DNA

Besides the 24 soil samples collected inside the preserved World War II site in Harbin, People's Republic of China (*1*), on two occasions—August 28th, 2019 (including the no. twenty-four soil sample from which the BA20200413YY strain was isolated) and June 20th, 2022—we also collected an additional 24 soil samples from 12 nearby sites within radii of 0.5 km, 3 km, and 5 km on July 12th, 2021. Each of the 12 additional sites underwent two samplings to acquire both surface and subsurface (10 cm depth) soil samples. The sampling orientation was not strictly constrained to north–south and east-west directions, due to the influence of nearby structures. The 24 soil samples collected from the surrounding area of the historical site were processed for DNA extraction using the DNeasy PowerSoil Pro kit (QIAGEN), and *B. anthracis* genomic DNA was detected using the DETECTR-*B. anthracis* system, following the same protocol used for the 24 soil samples collected within the historic site (*1*).

Isolation and Phenotypic Identification of Strain BA20200413YY

The BA20200413YY strain was isolated from a *B. anthracis* positive soil sample (Sample 24) (*1*) collected inside the historic site. The isolation was conducted as follows: 0.8g of soil was mixed with 4mL of PBS by vortexing to prepare a soil suspension in a biosafety cabinet in a BSL-3 laboratory. The *B. anthracis*-positive soil suspension was then inoculated onto a selective Polymyxin B-Lysozyme-EDTA-Thallous acetate (PLET) agar plate (Elite Media) using the streaking method and kept in the biosafety cabinet at room temperature. After ≈3 days, when colonies appeared on the plate, a single clone was selected for further culture on Columbia blood agar plates using the quadrant streaking method to obtain pure cultures. Plates with obvious hemolytic colonies were discarded, and colonies without obvious hemolysis were identified. An appropriate amount of a single clone colony was then boiled in 100µL of deionized water for 30 minutes, followed by centrifugation. The supernatant was used for RPA/CRISPR or PCR identification (*1*). Gram staining and API 50CHB-API 50CH (BioMérieux) biochemical identification were performed on positive colonies for further confirmation. The process of isolation and identification is outlined in Appendix Figure 4.

Whole-Genome Sequencing and Assembly

The DNA of the BA20200413YY strain was extracted using the DNeasy Blood & Tissue kit (QIAGEN, No. 69506). Whole-genome sequencing was conducted using the Illumina MiSeq and PacBio Sequel I platforms. The short-read sequencing data were processed using Trimmomatic (v0.38) (*2*) for adaptor trimming and read filtering (<Q20), while the long-read sequencing data was filtered based on read length (<1000 bp) using Filtlong (v0.2.1, https://github.com/rrwick/Filtlong). Subsequently, Unicycler (v0.4.9) (*3*) was used for hybrid assembly. The resulting assembly was manually corrected in combination with the Canu (v1.8) (*4*) assembly results to circularize the chromosome and obtain the complete genome.

All publicly available genomes of *B. anthracis* in NCBI databases (as of November 2022), including assemblies from the GenBank database and short-read sequencing data from the SRA database, were retrieved. The short-read sequencing data underwent trimming and filtering using Trimmomatic, followed by assembly using the SPAdes genome assembler (v3.13.0) (*5*). After excluding strains with assembly abnormalities or those that did not belong to *B. anthracis*, a final dataset of 1,553 genomes was obtained, which included BA20200413YY and 1,552 publicly available data (dataset is available in the Figshare repository at https://doi.org/10.6084/m9.figshare.26333143).

Antimicrobial Resistance Genes and Virulence Factors Detection

The complete genome of BA20200413YY was subjected to a blast analysis against the DNA sequences from the Comprehensive Antibiotic Resistance Database (CARD) (*6*) and the core dataset of the Virulence Factor Database (VFDB) (*7*) using BLASTn (v2.13.0) to identify antimicrobial resistance genes and virulence genes. Genes that met the criteria of an identity ≥90% and a coverage ≥90% were selected for further analysis.

SNP Calling and Phylogenetic Analysis

A dataset of 1,553 genomes was aligned to the chromosome of the reference *B. anthracis* str. 'Ames Ancestor' (Accession: NC_007530.2) using MUMmer (v3.23) (*8*) to identify core genome SNPs. SNPs located in repetitive regions, as identified by BLASTn and Tandem Repeat Finder (TRF, v4.07b), were excluded. A total of 11,967 SNPs were identified. The concatenated SNPs were used to construct a maximum likelihood (ML) tree using IQ-TREE (v1.6.6) (*9*) under the GTR+G model with a fast bootstrap value of 1000.

After excluding genetically modified or repeated sequencing strains with the same name, a total of 113 strains located in clade 5.2 were selected for a fine-scale analysis. Additionally, one strain from clade 5.1 (V770-NP-1R) was included as an outgroup, resulting in a final dataset of 114 strains (Appendix Table 3). Core genome SNPs were identified using assemblies, and

SNPs for strains with available raw sequencing data (except for BA111 and 34F2 with low sequencing depth) were validated using BWA (v0.7.12-r1039) (*10*) and GATK HaplotypeCaller (v4.2.4.0) (*11*). Only SNPs supported by at least 10 reads and with a minimum allele frequency of 90% were retained, resulting in a total of 1,615 SNPs. To investigate the phylogenetic relationships among the strains, a ML tree was constructed using IQ-TREE under the GTR+G model, with a bootstrap value of 100.

Indel Calling and Genomic Gain and Loss Analysis

We used BWA and GATK HaplotypeCaller to detect indels in the BA20200413YY strain, using the chromosome of str. 'Ames Ancestor' as the reference. The identified indels were compared with those of nine closely related strains to identify strain-specific indels. Furthermore, Prokka (v1.14.6) (*12*) was used for genome annotation of BA20200413YY and the nine closely related strains. Gene gain and loss events were then identified using Roary (v3.13.0) (*13*). The obtained results were validated using blast.

Plot of World Map

The world map was plotted using the maps and ggplot2 packages in R (v4.2.0).

References

1. Xu J, Bai X, Zhang X, Yuan B, Lin L, Guo Y, et al. Development and application of DETECTR-based rapid detection for pathogenic *Bacillus anthracis.* Anal Chim Acta. 2023;1247:340891. PubMed https://doi.org/10.1016/j.aca.2023.340891

2. Bolger AM, Lohse M, Usadel B. Trimmomatic: a flexible trimmer for Illumina sequence data. Bioinformatics. 2014;30:2114–20. PubMed https://doi.org/10.1093/bioinformatics/btu170

3. Wick RR, Judd LM, Gorrie CL, Holt KE. Unicycler: Resolving bacterial genome assemblies from short and long sequencing reads. PLOS Comput Biol. 2017;13:e1005595. PubMed https://doi.org/10.1371/journal.pcbi.1005595

4. Koren S, Walenz BP, Berlin K, Miller JR, Bergman NH, Phillippy AM. Canu: scalable and accurate long-read assembly via adaptive *k*-mer weighting and repeat separation. Genome Res. 2017;27:722–36. PubMed https://doi.org/10.1101/gr.215087.116

5. Bankevich A, Nurk S, Antipov D, Gurevich AA, Dvorkin M, Kulikov AS, et al. SPAdes: a new genome assembly algorithm and its applications to single-cell sequencing. J Comput Biol. 2012;19:455–77. PubMed https://doi.org/10.1089/cmb.2012.0021

6. Alcock BP, Raphenya AR, Lau TTY, Tsang KK, Bouchard M, Edalatmand A, et al. CARD 2020: antibiotic resistome surveillance with the comprehensive antibiotic resistance database. Nucleic Acids Res. 2020;48(D1):D517–25. PubMed

7. Liu B, Zheng D, Zhou S, Chen L, Yang J. VFDB 2022: a general classification scheme for bacterial virulence factors. Nucleic Acids Res. 2022;50(D1):D912–7. PubMed https://doi.org/10.1093/nar/gkab1107

8. Delcher AL, Salzberg SL, Phillippy AM. Using MUMmer to identify similar regions in large sequence sets. Curr Protoc Bioinformatics. 2003;Chapter 10:3. PubMed https://doi.org/10.1002/0471250953.bi1003s00

9. Nguyen LT, Schmidt HA, von Haeseler A, Minh BQ. IQ-TREE: a fast and effective stochastic algorithm for estimating maximum-likelihood phylogenies. Mol Biol Evol. 2015;32:268–74. PubMed https://doi.org/10.1093/molbev/msu300

10. Li H. Aligning sequence reads, clone sequences and assembly contigs with BWA-MEM. Quant Biol. 2013. https://doi.org/10.48550/arXiv.1303.3997

11. Van der Auwera GA, Carneiro MO, Hartl C, Poplin R, Del Angel G, Levy-Moonshine A, et al. From FastQ data to high confidence variant calls: the Genome Analysis Toolkit best practices pipeline. Curr Protoc Bioinformatics. 2013;43:10.1, 33. PubMed https://doi.org/10.1002/0471250953.bi1110s43

12. Seemann T. Prokka: rapid prokaryotic genome annotation. Bioinformatics. 2014;30:2068–9. PubMed https://doi.org/10.1093/bioinformatics/btu153

13. Page AJ, Cummins CA, Hunt M, Wong VK, Reuter S, Holden MT, et al. Roary: rapid large-scale prokaryote pan genome analysis. Bioinformatics. 2015;31:3691–3. PubMed https://doi.org/10.1093/bioinformatics/btv421

Appendix Table 1. Antimicrobial resistance genes and virulence genes identified in BA20200413YY*

Pos	Start	End	Gene ID	Gene Name	Type	Note
Chr	1494385	1495296	ARO:3003072	*mphL*	macrolide phosphotransferase	AMR
Chr	2327667	2328596	ARO:3000090	*bla1*	Bla β-lactamase	AMR
Chr	2933169	2933723	ARO:3005045	*satA*	streptothricin acetyltransferase	AMR
Chr	3216670	3217440	ARO:3004189	*bla2*	Bla β-lactamase	AMR
Chr	3778043	3778462	ARO:3005100	*fosB2*	fosfomycin thiol transferase	AMR
Chr	551812	555024	VFG016362	*hal*	Hal	VF
Chr	688462	690861	VFG016338	*inhA*	InhA (Exoenzyme)	VF
Chr	1239416	1241803	VFG016346	BAS_RS06430	InhA (Exoenzyme)	VF
Chr	1286136	1288449	VFG016354	*ilsA*	IlsA	VF
Chr	1769926	1771086	VFG016268	*nheA*	Nhe (Exotoxin)	VF
Chr	1771118	1772326	VFG016276	*nheB*	Nhe (Exotoxin)	VF
Chr	1772434	1773513	VFG016284	*nheC*	Nhe (Exotoxin)	VF
Chr	1863845	1865653	VFG049956	*asbA*	Petrobactin	VF
Chr	1865712	1867550	VFG049962	*asbB*	Petrobactin	VF
Chr	1867537	1868775	VFG049968	*asbC*	Petrobactin	VF
Chr	1868772	1869047	VFG049974	*asbD*	Petrobactin	VF
Chr	1869071	1870054	VFG049980	*asbE*	Petrobactin	VF
Chr	1870092	1870934	VFG049986	*asbF*	Petrobactin	VF
Chr	2042075	2046082	VFG040864	*essC*	T7SS	VF
Chr	2046210	2046482	VFG040865	*esxB*	T7SS	VF
Chr	2046566	2049242	VFG040866	BAS_RS10590	T7SS	VF
Chr	2051938	2052375	VFG040867	BAS_RS10600	T7SS	VF
Chr	2052432	2053847	VFG040868	*esxL*	T7SS	VF
Chr	2201122	2201898	VFG049993	*dhbA*	Bacillibactin	VF
Chr	2201925	2203124	VFG050004	*dhbC*	Bacillibactin	VF
Chr	2203137	2204753	VFG050015	*dhbE*	Bacillibactin	VF
Chr	2204785	2205672	VFG050026	*dhbB*	Bacillibactin	VF
Chr	2205704	2212861	VFG050037	*dhbF*	Bacillibactin	VF
Chr	3086138	3087676	VFG016216	*alo*	ALO (Exotoxin)	VF
pXO1	4042	6336	VFG000677	*pagA*	Anthrax toxin	VF
pXO1	9623	12052	VFG000676	*lef*	Anthrax toxin	VF
pXO1	148851	150809	VFG049919	*bslA*	BslA (Adherence)	VF
pXO1	164636	167038	VFG000678	*cya*	Anthrax toxin	VF
pXO1	169793	171220	VFG002040	AAD32423	AtxA (Regulation)	VF
pXO2	24031	25425	VFG000682	*capB*	Capsule	VF
pXO2	25440	25889	VFG000681	*capC*	Capsule	VF
pXO2	25901	27136	VFG000680	*capA*	Capsule	VF
pXO2	27319	28719	VFG000679	*dep/capD*	Capsule	VF

*AMR, antimicrobial resistance gene; VF, virulence factor.

Appendix Table 2. Lineage-specific and strain-specific variations associated with BA20200413YY*

Chr Position	Ref	Mut	Gene ID	Gene Name	Mut Type	Gene Product	Note
1064339	C	G	GBAA_RS05655	NA	nonsyn	YhgE/Pip domain-containing protein	lineage-specific SNPs
2045104	G	A	GBAA_RS10890	*essC*	nonsyn	type VII secretion protein EssC	lineage-specific SNPs
3601031	G	A	GBAA_RS19065	NA	nonsyn	NA	lineage-specific SNPs
5143657	G	T	GBAA_RS27565	NA	nonsyn	FMN-dependent NADH-azoreductase	lineage-specific SNPs
5157301	G	A	GBAA_RS27625	NA	nonsyn	TRIC cation channel family protein	lineage-specific SNPs
257312	C	T	GBAA_RS01500	NA	nonsyn	YdiK family protein	strain-specific SNPs
339229	C	T	GBAA_RS01850	NA	nonsyn	polysaccharide deacetylase family protein	strain-specific SNPs
552008	C	T	GBAA_RS03055	*yhbH*	nonsyn	sporulation protein YhbH	strain-specific SNPs
652649	C	T	GBAA_RS03460	*gerKA*	nonsyn	spore germination protein GerKA	strain-specific SNPs
897284	T	C	GBAA_RS04775	*sap*	nonsyn	S-layer protein Sap	strain-specific SNPs
1014486	C	T	---†	---	intergenic	---	strain-specific SNPs
1056704	C	T	GBAA_RS05625	NA	nonsyn	DUF6359 domain-containing protein	strain-specific SNPs
1819702	C	A	GBAA_RS09655	NA	nonsyn	ABC transporter ATP binding protein	strain-specific SNPs
2001530	C	T	GBAA_RS10685	NA	nonsyn	uroporphyrin-III C-methyltransferase	strain-specific SNPs
2195614	T	C	GBAA_RS11710	NA	nonsyn	SMC family ATPase	strain-specific SNPs
2949122	A	G	GBAA_RS15665	*hscC*	nonsyn	molecular chaperone HscC	strain-specific SNPs
3089423	G	A	GBAA_RS16360	NA	syn	short-chain fatty acid transporter	strain-specific SNPs
3623894	A	G	GBAA_RS19170	*truB*	nonsyn	tRNA pseudouridine(55) synthase TruB	strain-specific SNPs
3708907	G	A	GBAA_RS19570	*pyrB*	syn	aspartate carbamoyltransferase	strain-specific SNPs
3739955	A	G	GBAA_RS19715	*rsmH*	nonsyn	16S rRNA (cytosine(1402)-N(4))-methyltransferase RsmH	strain-specific SNPs
3966754	G	A	GBAA_RS21110	*cpdB*	nonsyn	bifunctional 2',3′-cyclic-nt 2'-phosphodiesterase/3′-nucleotidase	strain-specific SNPs
3967517	C	A	GBAA_RS21110	*cpdB*	nonsyn	bifunctional 2',3′-cyclic-nt 2'-phosphodiesterase/3′-nucleotidase	strain-specific SNPs
4607982	G	A	GBAA_RS24700	NA	nonsyn	response regulator transcription factor	strain-specific SNPs
4659870	T	G	GBAA_RS25030	NA	nonsyn	glucose-6-phosphate isomerase	strain-specific SNPs
4711453	C	A	---	---	intergenic	---	strain-specific SNPs
1623322	G	GT	GBAA_RS08705	NA	frameshift	antibiotic biosynthesis monooxygenase	strain-specific indels
1639581	T	TACACCAG	GBAA_RS08800	NA	frameshift	NA	strain-specific indels
1844892	A	AT	GBAA_RS09785	NA	frameshift	DUF3139 domain-containing protein	strain-specific indels
3551711	CA	C	GBAA_RS18845	NA	frameshift	DUF2515 domain-containing protein	strain-specific indels
4047346	G	GA	---	---	intergenic	---	strain-specific indels
4310159	C	CT	GBAA_RS23030	NA	frameshift	hypothetical protein	strain-specific indels

*Reference genome: the chromosome sequence of *B. anthracis* str. 'Ames Ancestor' (Accession: NC_007530.2)
†The symbol --- denotes variations located in intergenic regions, where Gene ID, Gene name, and Gene product are not available

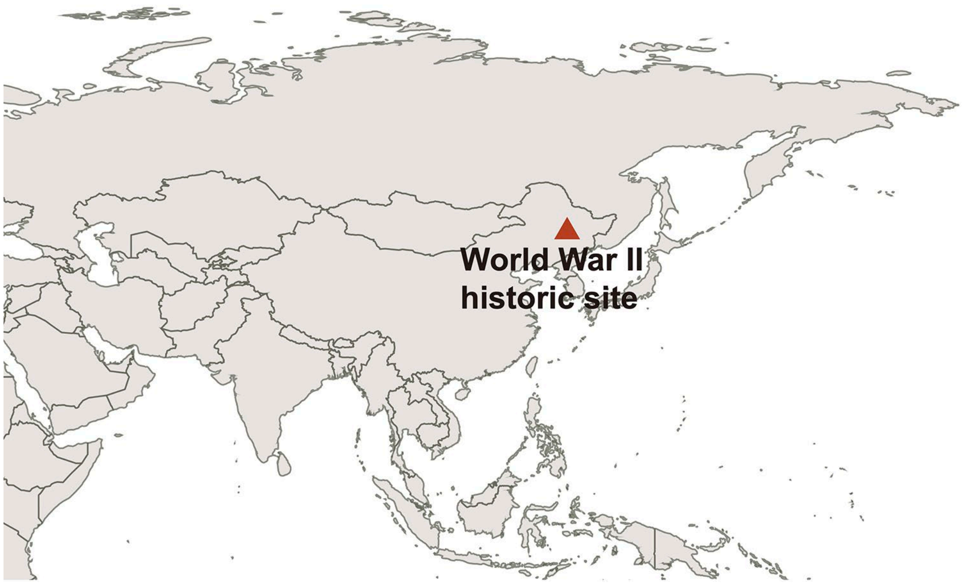

Appendix Figure 1. Location of the World War II laboratory remains.

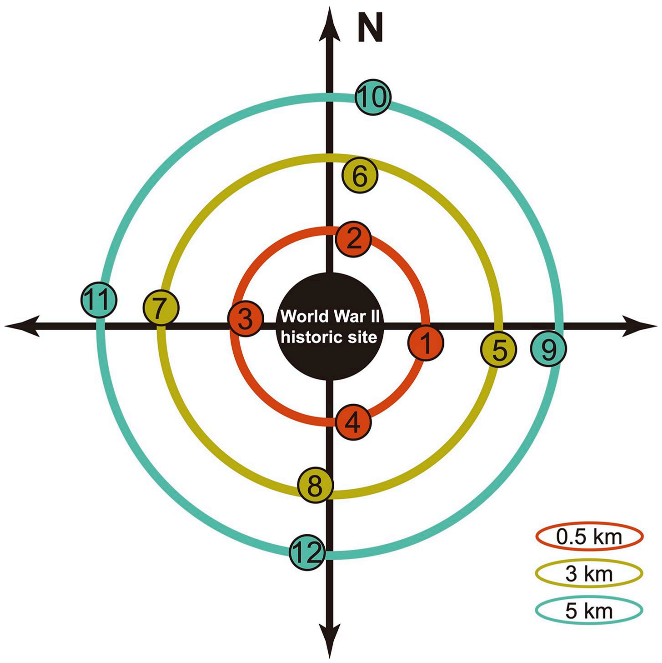

Appendix Figure 2. Schematic of sample collection sites: 24 previously reported samples from the historic site (Reference 5 in the main text) and 24 new samples from 12 additional sites located within 0.5 km, 3 km, and 5 km radii. Each of the 12 additional sites was sampled twice, including surface and subsurface (10 cm depth) soil samples. Sampling orientation was influenced by surrounding structures, resulting in deviations from strict north-south and east-west directions.

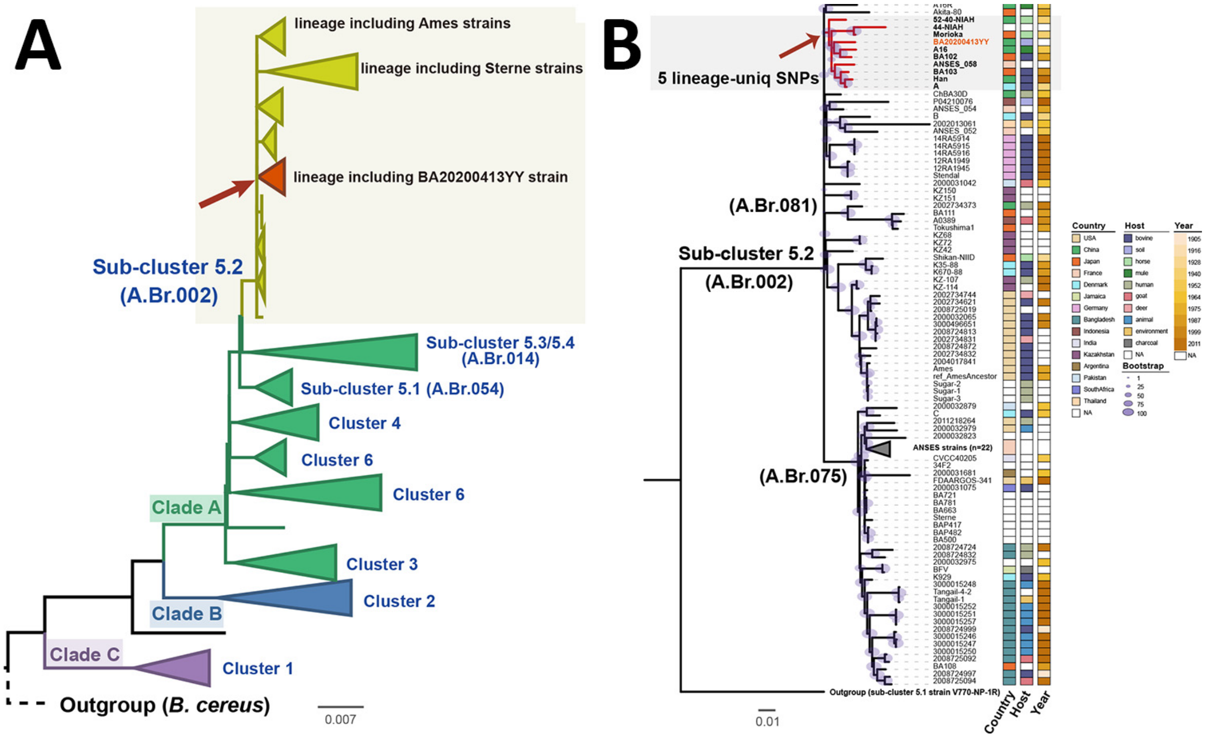

Appendix Figure 3. Phylogenetic analysis of *Bacillus anthracis* strains. A) Maximum likelihood (ML) tree of 1,553 *B. anthracis* strains based on 11,967 core genome SNPs, using strain Ames Ancestor chromosome sequence as reference. Branches were collapsed to improve visualization based on their clade and cluster designations. Bootstrap values are provided for main branches. The 3 main clades are color-coded. The subcluster 5.2, also known as A.Br.002 lineage, which includes strain BA20200413YY, is highlighted in yellow. The phylogenetic position of BA20200413YY is emphasized in red and marked by a red row. B) ML tree of subcluster 5.2 strains, including BA20200413YY, 112 selected public genomes from subcluster 5.2, and one strain from subcluster 5.1 as an outgroup. The sizes of the purple ellipses represent maximum bootstrap values. Three bars on the right depict sample source, host, and isolation time. Nine strains most closely related to BA20200413YY are highlighted by red branches.

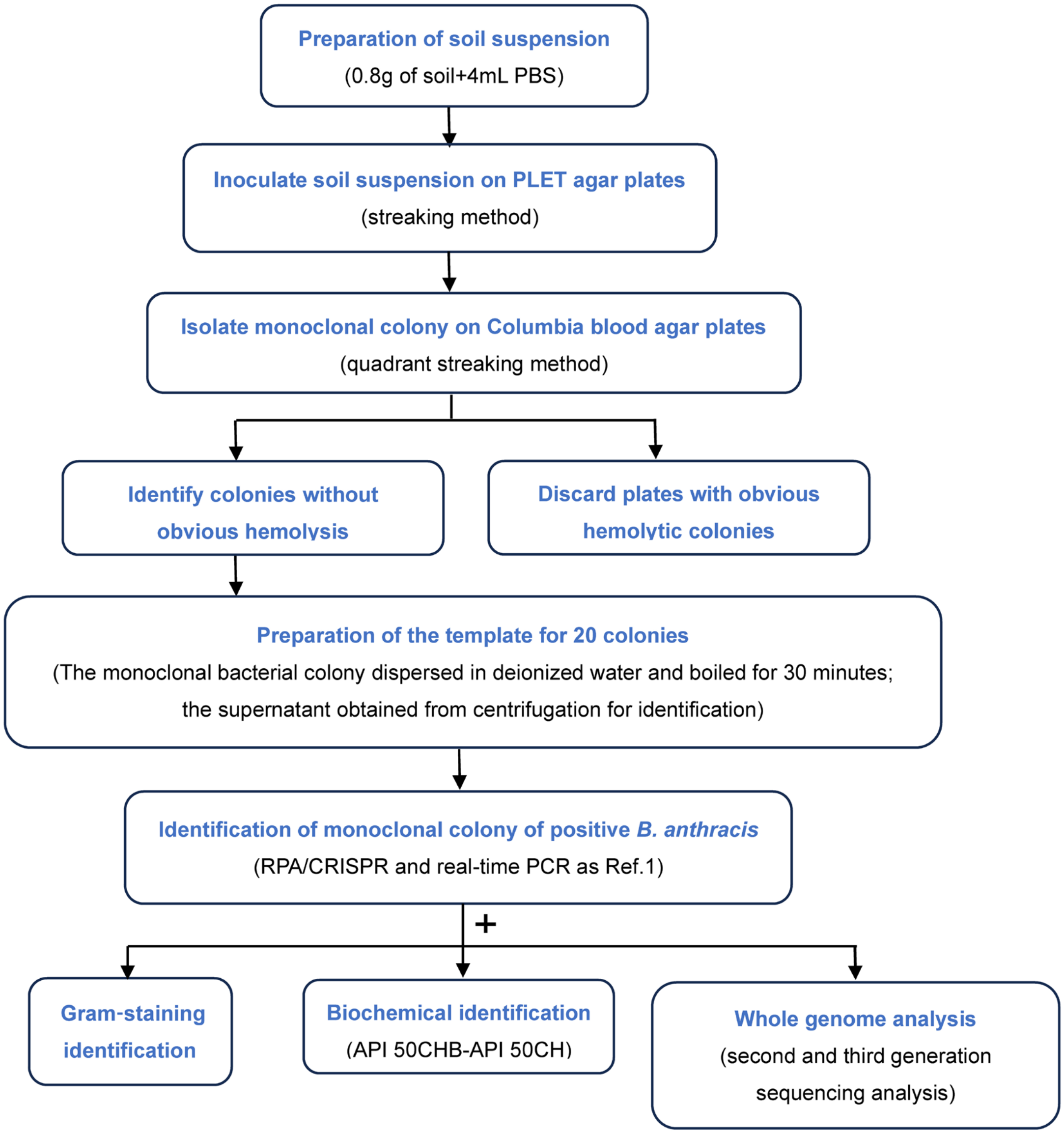

Appendix Figure 4. Pipeline for isolating and phenotypically identifying the *Bacillus anthracis* strain.
